# Maternal anemia and baby birth size mediate the association between short birth interval and under-five undernutrition in Ethiopia: a generalized structural equation modeling approach

**DOI:** 10.1186/s12887-022-03169-6

**Published:** 2022-02-28

**Authors:** Desalegn Markos Shifti, Catherine Chojenta, Elizabeth G. Holliday, Deborah Loxton

**Affiliations:** 1 Saint Paul’s Hospital Millennium Medical College, Addis Ababa, Ethiopia; 2 Centre for Women’s Health Research, School of Medicine and Public Health, University of Newcastle, New South Wales, Australia; 3 Centre for Clinical Epidemiology and Biostatistics, School of Medicine and Public Health, University of Newcastle, New South Wales, Australia

**Keywords:** Short birth interval, Anemia, Baby birth size, Stunting, Wasting, Underweight, Undernutrition, Mediation analysis, GSEM, Ethiopia

## Abstract

**Background:**

Studies assessing the association between short birth interval, a birth-to-birth interval of less than 33 months, and under-five undernutrition have produced inconclusive results. This study aimed to assess the relationship between short birth interval and outcomes of stunting, underweight, and wasting among children aged under-five in Ethiopia, and potential mediation of any associations by maternal anemia and baby birth size.

**Method:**

Data from the 2016 Ethiopia Demographic and Health Survey (EDHS) was used. Stunting, wasting, and underweight among children aged under-five were outcome variables. Generalized Structural Equation Modeling (GSEM) was used to examine associations between short birth interval and outcomes, and to assess hypothesized mediation by maternal anemia and baby birth size.

**Results:**

Significant associations between short birth interval and stunting (AOR = 1.49; 95% CI = 1.35, 1.66) and underweight (AOR = 1.43; 95% CI = 1.28, 1.61) were found. There was no observed association between short birth interval and wasting (AOR = 1.05; 95% CI = 0.90, 1.23). Maternal anemia and baby birth size had a significant partial mediation effect on the association between short birth interval and stunting (the coefficient reduced from *β* = 0.337, *p* < 0.001 to *β* = 0.286, *p* < 0.001) and underweight (the coefficient reduced from *β* = 0.449, *p* < 0.001 to *β* = 0.338, *p* < 0.001). Maternal anemia and baby birth size mediated 4.2% and 4.6% of the total effect of short birth interval on stunting and underweight, respectively.

**Conclusion:**

Maternal anemia and baby birth size were identified as mediators of the association between short birth interval and under-five undernutrition status. Policies and programs targeting the reduction of under-five undernutrition should integrate strategies to reduce maternal anemia and small baby birth size in addition to short birth interval.

## Background

According to the World Health Organization (WHO) recommendation, short birth interval is defined as a birth-to-birth interval of less than 33 months [1]. Short birth interval is more common among women in low- and middle-income countries [2] and Ethiopia has an estimated prevalence of 45.8% [3]. The determinants of short birth interval in Ethiopia, such as maternal occupation, wealth index, and regions, have been documented elsewhere [3]. Our previous studies [4, 5] also have documented the hotspot areas [4] and socioeconomic inequality [5] of short birth interval in Ethiopia.

Some of the adverse child health outcomes associated with short birth intervals are preterm birth [6, 7] low birth weight [6, 7] small size for gestational age [6] congenital anomalies [8, 9], and autism [10]. Similarly, miscarriage, preeclampsia, and premature rupture of membranes [11, 12], and maternal anemia [13, 14] are among the poor maternal health outcomes associated with short birth interval.

Previous studies assessing associations between short birth interval and under-5 child undernutrition have been inconclusive. Undernutrition, in our study, refers to stunting (short-for-age), underweight (thin-for-age), and wasting (thin-for-height) [15]. Some previous literature has documented the significant association between short birth interval and stunting [16–18], wasting [19], and underweight [20]. Other studies have found no significant associations reported between short birth interval and stunting [21, 22] and underweight [23]. In the previous studies [16–23], short birth interval was not defined according to the WHO recommendation, which is less than 33 months [1]. The limitation of some of the above-mentioned studies [18, 20, 21, 24–31] was that they did not use nationally representative data. Alternatively, several studies [24–28, 31–43] that have investigated determinants of undernutrition among children in Ethiopia did not consider short birth interval as a potential causal factor.

Other limitation of previous work is the inclusion of maternal anemia [16, 19] and baby birth size [19, 22] as confounders in estimating the association between short birth interval and child malnutrition. However, these factors are likely to lie on the causal pathway between short birth interval and undernutrition outcomes and are thus more likely to be mediators than confounders. That is, maternal anemia [13, 14] and baby birth size [6, 7] are health outcomes that can result from short birth interval (i.e., mediators), rather than being causes of short birth interval and child undernutrition (i.e., confounders). By definition, a confounder is a variable that has a direct causal effect on the main exposure variable and the outcome of interest [44, 45]. A mediator is intervening variables that lie along the causal pathway between the exposure/intervention and the outcome of interest [46]. Adjustment of mediators as confounders will under-estimate the causal effect of the variable of interest (short birth interval in this case) on the outcome variable (stunting, wasting, and underweight in this case) and may reduce the ability to identify the total causal effect of interest [45, 47]. The policy and program implication of investigating the mediation effect of maternal anemia and baby size is its ability to identify targets for interventions to prevent the development of child undernutrition.

Africa and Asia bear the greatest share of all forms of malnutrition [48, 49]. In Ethiopia, 38.0% of under-five children are stunted, 24% are underweight, and 10% are wasted [15]. One-fourth of child deaths in Ethiopia are associated with malnutrition [50]. As a result, the Ethiopian government developed the *Seqota* Declaration aiming to end malnutrition, particularly stunting, by 2030 [51, 52]. It is also known that one of the 2030 agendas for Sustainable Development Goals (SDGs) is linked with ending all forms of malnutrition (i.e., SDG 2, target 2.1.1) [53, 54]. Nevertheless, undernutrition among children remains an urgent concern in Ethiopia [55], requiring the identification of its multifactorial predictors to make an informed decision and meet the above-mentioned goals [51–53].

To the best of our knowledge, no previous study has investigated the mediation effect of maternal anemia and baby birth size in the association between short birth interval and under-five undernutrition (i.e., the direct and indirect causal pathway). This study aimed to assess the mediation effect of maternal anemia and baby birth size in the association between short birth interval and under-five malnutrition; stunting, underweight, and wasting. The findings of this study will help policy makers and program planners consider the effect of short birth interval and potential mediators in combating undernutrition in Ethiopia.

## Methods

### Data source, design, and sample size

Data from the 2016 Ethiopia Demographic and Health Survey (EDHS) were used in this study. The EDHS is a nationally representative survey in Ethiopia and has been conducted every 5 years since 2000. The 2016 EDHS collected data on the nutritional status of children by measuring the weight and height of children under age 5 in all sampled households and comparing these to international standards [15]. Weight was measured with an electronic mother-infant scale (SECA 878 flat) designed for mobile use. Height was measured with a measuring board (Shorr Board®). Children younger than age 24 months were measured lying down on the board (recumbent length) while standing height was measured for the older children. Children’s height/length, weight, and age data were used to calculate three indices: height-for-age, weight-for-height, and weight-for-age. The DHS data were compared to the NCHS/CDC/WHO international reference standards for height-for-age, weight-for-age, and weight-for-height. Further information regarding the survey methodologies and measurement of nutritional status is presented in the full EDHS report [15]. Since short birth interval was the main exposure variable, the current study included women who had reported at least two live births during the five years preceding the survey. Accordingly, 7,090 women were included for analyses of stunting, 7,154 for wasting, and 7,233 for underweight. Respondents with missing data for height-for-age ($n$=1,358), weight-for-height ($n$= 1,294), and weight-for-age ($n$= 1,215) of their child were excluded from the analysis.

### Measurement and variables

#### Outcome variables

Under-five undernutrition represented by stunting (a height-for-age Z-score below minus two standard deviations (-2 SD) from the median of the reference population), wasting (a weight-for-height Z-score below minus two standard deviations (-2 SD) from the median of the reference population), and underweight (a weight-for-age Z-score below minus two standard deviations (-2 SD) from the median of the reference population) were the outcome variables [15].

#### Exposure variables

Short birth interval, defined as a birth-to-birth interval of less than 33 months [1], was the exposure variable in this study. Women’s birth interval data were collected by extracting dates of birth for their biological children from the children’s birth/immunization certificate, and/or asking the mother to provide dates of birth for their children. When both sources of data were available, the accuracy of documenting dates of birth in birth/immunization certificates was cross-checked with the information provided by mothers. This resolved discrepancies such as the documented birth date representing the date when the birth was recorded, rather than the actual birth date. When children’s birth/immunization certificates were not available, information regarding children’s date of birth was obtained from their mothers. The EDHS presented birth interval data in months. Detailed description regarding birth interval data collection is also provided elsewhere [4, 56].

#### Mediators

Maternal anemia and birth size were the two sequential mediators considered in this analysis. Women’s blood samples were drawn from a drop of blood taken from a finger prick and collected in a microcuvette. Hemoglobin analysis was carried out on-site using a battery-operated portable HemoCue analyser [15, 57, 58]. The hemoglobin level was adjusted for cigarette smoking and altitude in enumeration areas above 1,000 m. Anemia was defined as per the WHO recommendation, which is hemoglobin level less than 12.0 g/deciliter for pregnant women and 11 g/deciliter for non pregnant women [15, 59, 60]. In the 2016 EDHS, information on birth weight was collected by either a written record or maternal estimation. The current study used maternal estimated baby birth size as a proxy indicator for birth weight. All mothers who had given birth during the five years preceding the survey were asked to retrospectively classify their babies’ sizes at birth as ‘very large’, ‘larger than average’, ‘average’, ‘smaller than average’ or ‘very small’. This estimate was used because objectively measured birth weight data were not available for most (86%) newborns in Ethiopia [15]. Implications of this are discussed further in the Discussion section. The maternal estimated baby birth size was thus the only means of measuring birth size for the 86% of newborns with unknown birth weight, in a manner consistent with that of children (14%) whose birth weight was collected in the 2016 EDHS. In the current study, a baby’s birth size was coded as a binary variable. Very large, larger than average, and average responses were coded as ‘average or above average, and smaller than average and very small responses were coded as ‘small’.

### Covariates

Maternal age, maternal education (no formal education, primary, secondary +), maternal occupation (not working, working), household wealth (poorest, poorer, middle, richer, richest), place of residence (urban/rural), region (Tigray, Afar, Amhara, Oromia, Somalia, Benishangul-Gumuz, Southern Nations, Nationalities, and People’s Region [SNNPR], Gambella, Harari, Addis Ababa, Dire Dawa), and total children ever born (1–2, 3–4, 5 +) were considered as potential covariates (see Table 1). These covariates were selected after reviewing relevant literature [3, 16–23]

**Table 1:** The weighted distribution of short birth interval and respondents background characteristics by nutritional status, EDHS 2016

**Variable**	**Stunting** (*N* = 7,090)		***P*-value**	**Wasting** (*N* = 7,154)		***P*-value**	**Underweight** (*N* = 7,233)		***P*-value**
	**No (%)**	**Yes (%)**		**No (%)**	**Yes (%)**		**No (%)**	**Yes (%)**	
Maternal age at the birth of the index child (in years)									
≤ 24	634 (12.2)	310 (11.3)	0.002	802 (11.8)	142 (12.2)	0.070	721 (12.2)	241 (10.7)	0.741
25–29	1360 (31.6)	839 (31.0)		1951 (31.3)	269 (29.7)		1644 (31.4)	605 (31.0)	
30–34	1219(27.5)	680 (26.8)		1691 (27.5)	224 (26.0)		1430 (27.0)	507 (28.1)	
≥ 35	1240 (28.7)	808 (30.9)		1819 (29.4)	256 (32.1)		1532 (29.4)	553 (30.2)	
Maternal education									
No formal education	3019 (70.4)	2030 (78.1)	< 0.001	4416 (73.1)	702 (78.3)	< 0.001	3629 (70.8)	1532 (81.5)	< 0.001
Primary	1032 (23.7)	520 (20.0)		1403 (22.6)	147 (18.1)		1266 (24.2)	317 (16.4)	
Secondary +	402 (5.9)	87 (1.9)		444 (4.3)	42 (3.6)		432 (5.0)	57 (2.1)	
Maternal occupation									
Not employed	3240 (73.4)	1882 (72.1)	0.203	4496 (73.1)	679 (72.2)	< 0.01	3833 (72.7)	1398 (73.9)	0.243
Employed	1213 (26.6)	755 (27.9)		1767 (26.9)	212 (27.8)		1494 (27.3)	508 (26.1)	
Wealth									
Poorest	1589 (21.8)	1094 (28.2)	< 0.001	2284 (23.6)	461 (33.2)	< 0.001	1843 (22.5)	913 (31.4)	< 0.001
Poorer	730 (22.4)	564 (26.4)		1160 (24.2)	141 (22.2)		914 (23.0)	400 (27.1)	
Middle	647 (21.7)	397 (21.1)		935 (21.3)	115 (22.5)		795 (21.5)	265 (20.4)	
Richer	613 (19.3)	302 (16.1)		829 (18.5)	76 (12.1)		758 (19.6)	167 (12.6)	
Richest	874 (14.8)	280 (8.2)		1055 (12.4)	98 (10.0)		1017 (13.5)	161 (8.5)	
Number of children ever born									
≤ 2	726 (14.2)	320 (12.8)	< 0.001	937 (14.0)	104 (9.6)	< 0.05	861 (14.4)	205 (10.9)	< 0.001
3–4	1569 (34.8)	906 (31.9)		2175 (33.5)	302 (35.2)		1858 (34.0)	667 (33.7)	
≥ 5	2158 (51.0)	1411 (55.3)		3151 (52.5)	485 (55.2)		2608 (51.6)	1034 (55.4)	
Residence									
Urban	816 (10.9)	268 (6.2)	< 0.001	961 (9.0)	122 (9.7)	0.198	933 (9.9)	171 (6.1)	< 0.001
Rural	3637 (89.1)	2369 (93.8)		5302 (91.0)	769 (90.3)		4394 (90.1)	1735 (93.9)	
Region									
Tigray	420 (6.1)	301 (6.5)	< 0.001	634 (6.2)	85 (7.3)	< 0.001	544 (6.2)	184 (6.3)	< 0.001
Afar	371(1.0)	282 (1.0)		541 (0.9)	131 (1.7)		409 (0.8)	266 (1.4)	
Amhara	388 (16.9)	346 (24.1)		658 (19.6)	71 (18.6)		523 (18.2)	218 (23.7)	
Oromia	730(46.2)	414 (41.8)		1024 (44.3)	125 (47.8)		905 (45.7)	258 (41.1)	
Somali	721(5.3)	273 (3.0)		813 (4.0)	209 (10.0)		744 (4.2)	272 (5.1)	
Benishangul-Gumuz	344 (1.0)	272 (1.2)		550 (1.0)	67 (1.1)		405 (1.0)	224 (1.6)	
SNNPR^a^	551(20.6)	356 (21.2)		852 (21.7)	54 (12.0)		721 (21.3)	206 (19.6)	
Gambella,	323 (0.3)	114 (0.1)		382 (0.2)	67 (0.3)		349 (0.2)	90 (0.2)	
Harari	240 (0.2)	110 (0.2)		314 (0.2)	39 (0.2)		281 (0.2)	78 (0.2)	
Addis Ababa	191 (2.1)	30 (0.5)		211 (1.6)	9 (0.6)		213 (1.9)	12 (0.4)	
Dire Dawa	174 (0.3)	139 (0.4)		284 (0.3)	34 (0.4)		233 (0.3)	98 (0.4)	
Maternal anemia									
No	2831 (70.5)	1609 (67.1)	< 0.05	3947 (69.2)	514 (67.8)	< 0.01	3393 (69.5)	1124 (67.8)	< 0.001
Yes	1512 (29.5)	959 (32.9)		2157 (30.8)	359 (32.2)		1794 (30.5)	735 (32.2)	
Missing	110	69		159	18		140	47	
Birth size									
Average or above	3355 (76.7)	1801 (69.3)	< 0.001	4643 (74.7)	565 (67.3)	< 0.001	4028 (76.7)	1234 (64.8)	< 0.001
Small size	1098 (23.3)	836 (30.7)		1620 (25.3)	326 (32.7)		1299 (23.3)	672 (35.2)	
Short birth interval									
No	2478 (58.7)	1246 (50.8)	< 0.001	3327 (55.7)	410 (54.4)	< 0.001	2950 (57.3)	843 (50.3)	< 0.001
Yes	1975 (41.3)	1391 (49.2)		2936 (44.3)	481 (45.6)		2377 (42.7)	1063 (49.7)	

^a^*SNNPR* Southern Nations, Nationalities, and Peoples' Region, *EDHS* Ethiopia Demographic and Health Survey

### Data analysis

Descriptive statistics (frequency with percent) were computed to describe the outcome (stunting, wasting, and underweight) by the respondents’ characteristics. Pearson’s chi-squared tests were used to assess differences in stunting, wasting, and underweight frequencies by respondents’ characteristics. Sampling weight was considered to adjust for the non-proportional allocation of the sample to different regions, to their urban and rural areas, and the possible differences in response rates. Details about the weighting procedure can be found in the EDHS report [15]. Multivariable logistic regression analysis was used to assess the independent association between short birth interval and stunting, wasting, and underweight. Variables listed under the covariates above were included as potential confounders. Short birth interval showing a significant association with outcomes at a *p*-value of < 0.05 in the multivariable logistic regression analysis were considered to test whether the hypothesized mediators (maternal anemia and baby birth size) mediated the observed relationships using mediation analysis. This is because, first, there has to be a significant association between the main exposure variable (short birth interval in this case) and outcomes (i.e., stunting, underweight, and wasting in this case) to be mediated to further examine for the mediation effect of the potential mediators (maternal anemia and baby birth size in this case) [61]. Then, Generalized Structural Equation Modeling (GSEM) was used to test the mediation effect of the potential sequential mediators (i.e., maternal anemia and baby birth size) on under-five undernutrition. The mediation analysis was performed using Stata *‘gsem’* command. First, the initial path models were fitted using exposure variable (i.e., short birth interval), potential mediators (i.e., maternal anemia and baby birth size), and the outcomes (i.e., stunting, wasting, and underweight, each separately). This was done to assess crude associations between the above-mentioned variables. Second, after controlling for potential confounders, the full mediation analysis models were fitted for each child undernutrition outcome separately. Each outcome variable was a binary variable analyzed assuming a Bernoulli response distribution and logit link function. Mediation can be either complete or partial [44, 62–64]. In complete mediation, the entire (or total) effect of an exposure variable (i.e., short birth interval) on an outcome variable (i.e., stunting, wasting, and underweight) is transmitted through one or more mediators (i.e., maternal anemia and baby birth size in this case). Thus, the exposure variable has no direct effect on the outcome variables; its entire effect is indirect. In partial mediation, an exposure variable has both direct and indirect effects on the outcome variables. The direct effect is not mediated, whereas the indirect effect is transmitted through one or more mediator variables. Mediators can also be classified as single and multiple (and sequential) [62, 65]. A single mediator is considered when there is only one variable in the causal pathway between exposure and outcome variable. Multiple mediators refer to when more than one mediator variables operate jointly at the same stage in a causal model. Thus, there will be several indirect effects linking the exposure variable to the outcome variable. When the indirect effect of an exposure variable on the outcome variable operates through a chain of mediator variables, it refers to sequential mediators. For instance, maternal anemia and baby birth size, in the current study, could be considered as the sequential mediators. In this analysis, the indirect effects were estimated using the product-of-coefficients test [66, 67]. For a variable with missing data such as maternal anemia, a complete case analysis was performed with the assumption of missing completely at random. Stata *‘nlcom’* command was used to estimate the direct, indirect, total effects of short birth interval on child malnutrition. A *p*-value of < 0.05 was used to declare statistical significance. Statistical analysis was performed using Stata version 14 statistical software *(StataCorp. Stata Statistical Software: Release 14. College Station, TX: StataCorp LP. 2015).*

## Results

### Participant characteristics

The majority of stunting (78.1%), wasting (78.3%), and underweight (81.5%) were documented among children of women with no formal education. Similarly, 72.1% of stunting, 72.2% of wasting, and 73.9% of underweight were experienced by children of unemployed women. The prevalence of stunting, wasting, and underweight were higher (> 90.0% for each) among rural residents. About half of stunting, wasting, and underweight were among children born after a short birth interval (Table 1).

### The association between short birth interval and under-five undernutrition status

After conditioning on the potential confounders, significant associations between short birth interval and stunting (AOR = 1.49; 95% CI = 1.35, 1.66) and underweight (AOR = 1.43; 95% CI = 1.28, 1.61) were found. There was no significant association between short birth interval and wasting (AOR = 1.05; 95% CI = 0.90, 1.23) (Table 2).

**Table 2:** The associations between short birth interval and undernutrition of children in Ethiopia, EDHS 2016

Variable	Stunting		Wasting		Underweight	
	COR (95% CI)	AOR (95% CI)^a^	COR (95% CI)	AOR (95% CI) ^a^	COR (95% CI)	AOR (95% CI) ^a^
Short birth interval						
No	Ref	Ref	Ref	Ref	Ref	Ref
Yes	1.40 (1.27, 1.54)*	1.49 (1.35, 1.66)*	1.33 (1.15, 1.53)*	1.05 (0.90, 1.23)	1.56 (1.41, 1.74)*	1.43 (1.28, 1.61)*

*EDHS* Ethiopia Demographic and Health Survey, *COR* Crude Odds Ratio, *AOR* Adjusted Odds Ratio, *CI* Confidence Interval, *Ref* reference group; *P value < 0.001; **P value < 0.01; ***P value < 0.5; ^a^The models were adjusted for maternal age at the birth of the index child, maternal education, maternal occupation, wealth, place of residence, regions, and number of children ever born

### Mediation analysis

**Fig. 1:**
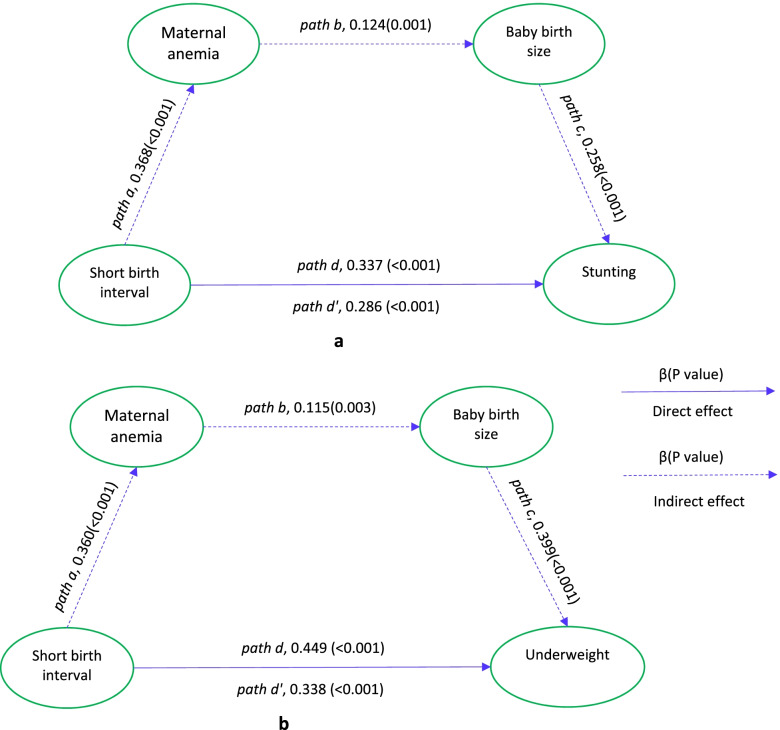
**a** and **b** Mediation effect of maternal anemia and baby birth size on the relationship between short birth interval and stunting (**a**) and underweight (**b**)

Table 3 and Fig. 1a and b illustrate findings from the mediation analysis. Short birth interval was significantly associated with stunting (*path d*, *β* = 0.337, *p* < 0.001). Significant associations were also found between short birth interval and maternal anemia (*path a*, *β* = 0.368, *p* < 0.001), maternal anemia and baby birth size (*path b*, *β* = 0.124, *p* = 0.001), and baby birth size and stunting (*path c*, *β* = 0.258, *p* < 0.001). After conditioning on maternal anemia and baby birth size, the coefficient for short birth interval reduced in magnitude from *path d*, *β* = 0.337, *p* < 0.001 to *path d’*, *β* = 0.286, *p* < 0.001 (Fig. 1a). This finding indicated that the effect of short birth interval on stunting was partially mediated by maternal anemia and baby birth size. The sequential mediators, maternal anemia and baby birth size, mediated 4.2% of the total effect of short birth interval on stunting.

**Table 3:** Associations between exposure, sequential mediators and outcome variables, EDHS 2016

**Models**		***β***^**a**^** (95% CI)**	***P* value**
Stunting	Short birth interval and maternal anemia^†^	0.368 (0.267, 0.469)	< 0.001
	Maternal anemia and baby birth size ^††^	0.124 (0.048, 0.201)	0.001
	Birth size and stunting^†††^	0.258 (0.148, 0.367)	< 0.001
	Short birth interval and stunting^u^	0.337 (0.240, 0.434)	< 0.001
	Short birth interval and stunting^a^	0.286 (0.185, 0.387)	< 0.001
Underweight	Short birth interval and maternal anemia^†^	0.360 (0.261, 0.460)	< 0.001
	Maternal anemia and baby birth size ^††^	0.115 (0.039, 0.190)	0.003
	Birth size and underweight^†††^	0.399 (0.283, 0.515)	< 0.001
	Short birth interval and underweight^u^	0.449 (0.342, 0.553)	< 0.001
	Short birth interval and underweight^a^	0.338 (0.228, 0.448)	< 0.001

^a^The models were adjusted for maternal age at the birth of the index child, maternal education, maternal occupation, wealth, place of residence, regions, and number of children ever born

^u^The effect of short birth interval on stunting and underweight, respectively, without adjusting for maternal anemia and baby birth size

^a^The effect of short birth interval on stunting and underweight, respectively, adjusting for maternal anemia and birth size

^†,^^††,†††^The value of *β*’s and *P* values were different in the two models because the models were adjusted for different forms of child undernutrition

There was a significant association between short birth interval and underweight (*path d*, *β* = 0.449, *p* < 0.001). Significant associations were also found between short birth interval and maternal anemia (*path a*, *β* = 0.360, *p* < 0.001), maternal anemia and baby birth size (*path b*, *β* = 0.115, *p* = 0.003), and baby birth size and underweight (*path c*, *β* = 0.399, *p* < 0.001). After conditioning on maternal anemia and baby birth size, the coefficient for short birth interval reduced in magnitude from *path d*, *β* = 0.449, *p* < 0.001 to *path d’*, *β* = 0.338, *p* < 0.001 (Fig. 1b). This reduction in the magnitude of the coefficient illustrated that maternal anemia and baby birth size partially mediated the association between short birth interval and underweight. Maternal anemia and baby birth size mediated 4.6% of the total effect of short birth interval on underweight.

Since there was no significant association to be mediated between short birth interval and wasting (AOR = 1.05; 95% CI = 0.90, 1.23; as presented in Table 2), the mediation effect of maternal anemia and baby birth size was not assessed.

## Discussion

This study aimed to assess the relationship between short birth interval and stunting, underweight, and wasting among children aged under-five in Ethiopia, and potential mediation of any associations by maternal anemia and baby birth size. To our knowledge, no study, to date, has examined the mediation effect of maternal anemia and baby birth size on the association between short birth interval and under-five undernutrition. The study showed significant associations between short birth interval and stunting and underweight. Maternal anemia and baby birth size had significant partial mediation effects on the relationship between short birth interval and stunting and underweight. The evidence from this study will help policy makers and program planners design a multifaceted approach to reduce child undernutrition in Ethiopia.

Our study showed short birth interval was associated with stunting and underweight. Previous studies also reported similar findings regarding the association between short birth interval and stunting [16–18, 68] and underweight [20, 68, 69]. The associations between short birth interval and stunting and underweight could be attributed to the increased risk of intrauterine growth retardation [70], inappropriate complementary feeding [71], poor dietary diversity [72, 73], and inadequate minimum meal frequency [74] associated with short birth interval. Moreover, from a women's perspective, the perception of being undernourished by women with a short birth interval may influence their infant feeding choices, such as the duration and frequency of breastfeeding [70]. These choices could then influence the child’s nutritional status via direct effects attributable to nutrient intake and indirect effects attributable to morbidity.

The current study illustrated that maternal anemia and baby birth size mediated the association between short birth interval and stunting as well as underweight. The most common hypothesis for adverse maternal and child outcomes, such as undernutrition, secondary to short birth interval is maternal folate depletion [75, 76]. This hypothesis posits that a short birth interval gives women insufficient time to recover from folate requirements during pregnancy [77]. It is known that folate depletion can expose women to anemia, resulting from ineffective erythropoiesis [78]. Subsequently, maternal anemia could result in low birth weight [79–81]. Finally, low birth weight, in turn, could affect the development of stunting [21, 82–84] as well as underweight [84, 85] among children. This finding could imply the need to prevent a short birth interval as well as maternal anemia to prevent low baby birth size and their associated undernutrition.

In this study, short birth interval was not significantly associated with wasting. This finding is not consistent with the finding of a previous study conducted in Ethiopia [19], where a significant association between short birth interval and wasting was reported. The discrepancy could be due to the difference, first, in the study population where the previous study [19] included women who had given birth once. These women were not eligible to provide birth interval information and the result could have been different if they were excluded from the study. Including the above-mentioned non-eligible women may also obscure the true effect of birth interval on wasting. Second, unlike the current study, the categorization of birth interval data (i.e., first birth, < 24 months, 24–47 months, and ≥ 48 months) in the previous study [19] was not according to the WHO recommendation [1].

The key strength of our study was the application of GSEM, a robust statistical technique, to assess the mediation effect of maternal anemia and baby birth size in the association between short birth interval and child undernutrition. Using data from a large sample size and nationally representative survey are also another strength of the current study. This study has also a few limitations. First, the use of observational data may limit the establishment of a causal association between short birth interval, mediators, and undernutrition status of under-five children. Second, although studies [86, 87] from developing countries recommended the use of baby birth size as a proxy for birth weight, it should be considered with precaution while interpreting the findings of this study. This is because it could be influenced by societal and contextual factors. Our study, however, considered maternal and contextual characteristics, such as educational level, wealth status, place of residence, region, and other variables detailed under the covariates section, as a potential confounder in its analysis. In the 2016 EDHS, 28% of births were delivered by a skilled provider, and information on birth weight was obtained for only 14% of births [15]. In contrast, information on baby birth size was collected for all children included in the survey. Hence, our study used maternal estimated baby birth size as a proxy indicator of birth weight. Under some circumstances, such as in the absence of children’s birth/immunization certificates and inconsistency in information regarding children’s date of birth between the one documented in the above-mentioned certificates and those obtained from the maternal response, information regarding children’s date of birth was obtained from their mothers. This information may be prone to recall bias. The timing of occurrence of maternal anemia should also be carefully considered while interpreting the findings of the study.

## Conclusion

There were statistically significant associations between short birth interval and stunting and underweight. This study also revealed that the association between short birth interval and stunting and underweight were partially mediated by the sequential mediators; maternal anemia and baby birth size. Policies and programs targeting the reduction of under-five undernutrition (stunting and underweight) should integrate strategies to reduce maternal anemia and small baby birth size in addition to the short birth interval. Health care providers should create awareness of the adverse effect of short birth interval on children's nutritional status. We also recommend women space births at least 33 months. Expanding postpartum contraception could help women prevent short birth interval. Longitudinal data is required to better estimate the causal effect of short birth interval and its associated mediators on child undernutrition.

## Data Availability

The dataset is available from The DHS Program repository at the following link: https://www.dhsprogram.com/data/dataset/Ethiopia_Standard-DHS_2016.cfm?flag=0.
